# Novel multiport robotic systems versus da vinci multiport robotic system in robot-assisted partial nephrectomy: a systematic review and meta-analysis of surgical and oncological outcomes

**DOI:** 10.1007/s11701-026-03277-w

**Published:** 2026-03-05

**Authors:** Francesco Rossi, Maria Chiara Sighinolfi, Filippo Gavi, Marco Montesi, Daniele Fettucciari, Simone Assumma, Enrico Panio, Ela Patel, Francesco Pio Bizzarri, Simona Presutti, Giovanni Battista Filomena, Seyed Koosha Moosavi, Or Schubert, Antonio Silvestri, Giuseppe Pallotta, Pierluigi Russo, Filippo Maria Turri, Riccardo Bientinesi, Mauro Ragonese, Nazario Foschi, Angelo Totaro, Emilio Sacco, Bernardo Rocco

**Affiliations:** 1 Department of Urology, Fondazione Policlinico Universitario Agostino Gemelli IRCCS, Rome, Italy; 2 Department of Medicine and Translational Surgery, Università Cattolica Del Sacro Cuore, Rome, Italy; 3 Stanford University, Stanford, CA USA; 4 Department of Urology, Ospedale Isola Tiberina – Gemelli Isola, Rome, Italy

**Keywords:** Robotic surgery, Da Vinci surgical system, Robot-assisted partial nephrectomy, Comparative studies

## Abstract

Robotic surgery is a game-changing innovation in urology. Novel multiport robotic systems’s (MRS) benefits and limitations in renal surgery when compared to Da Vinci MRS need to be fully understood. This study aims to identify variations from the well-known Da Vinci MRS in terms of surgical, oncological, and functional outcomes in patients undergoing robot-assisted partial nephrectomy (RAPN). Our systematic review and meta-analysis screened Pubmed, Web of Science, and Scopus databases, analyzing data from eighteen papers. Of them, 6 studies compared RAPN outcomes in patients between the novel MRS and Da Vinci MRS. Surgical outcomes (surgical times, estimated blood loss, length of hospital stay, Clavien-Dindo grade > 2 complications rate) and oncological outcomes (positive surgical margins rate and trifecta achievement rate) were analyzed using a Sidik-Jonkman method in a random-effects model. The abovementioned eighteen studies involved and included 1204 patients (631 novel MRS and 573 Da Vinci). Six of them were comparative studies between novel MRS and Da Vinci. Meta-analysis between novel MRS and Da Vinci revealed statistically significant differences in terms of docking time (Mean Difference 1.95; 95% CI 0.41–3.5; I^2^ 98.9%; *p* = 0.01) and console time (Mean Difference 16.72; 95% CI 5.1–28.4; I^2^ 67.7%; *p* < 0.01). No other variations between the two systems were shown in terms of oncological or perioperative results. The present systematic review and meta-analysis demonstrates similar surgical and clinical outcomes for RAPN conducted with the novel MRS and Da Vinci robotic platform, despite the small number of included studies. Novel MRS could be used in resource-limited hospital settings as alternative to Da Vinci MRS.

**Supplementary Information:**

The online version contains supplementary material available at 10.1007/s11701-026-03277-w.

## Introduction

Robot-assisted surgery emerged as a game-changing technique in urology thanks to several technological changes in the past few decades. Its advantages include improved precision, reduced hospitalization times, enhanced surgical field visualization, and revolutionizing surgical approach to renal masses [1]. Partial nephrectomy rapidly became one of the most advantageous procedures in robotic surgery; its precision, dexterity, and relatively short learning curve allowed the diffusion of the partial over radical approach [2]. Despite that, the adoption of robotic surgery has been historically restricted to high-volume and economically stable hospitals due to its considerable costs, particularly in resource-limited settings [1, 3].

Since its introduction in clinical practice, the Da Vinci Multiport Robotic System (MRS) (Intuitive Surgical Inc., Sunnyvale, CA, USA) has been the cornerstone. With Intuitive’s patent expiring in 2019, several MRS have been developed in many Countries. The Senhance robotic system, followed by REVO-I, Versius, Avatera, Hinotori, KangDuo, Toumai, Dexter, and Hugo robot-assisted surgery (RAS) systems emerged as competitors of the Da Vinci robotic systems in the last decade [4]. This large selection of novel MRS aims to increase access to robotic surgery while maintaining the same advantageous properties of the Da Vinci system [5]. Notably, emerging robotic platforms exhibit both unique and familiar features, such as varying console types (immersive vs. open) and patient cart configurations (single boom vs. multi-cart), distinguishing them from the Da Vinci system. Additionally, the available instruments and their capabilities can differ between platforms, influencing their suitability for specific procedures [6].

The use of new systems for partial nephrectomy has been addressed in recent years. However, safety and feasibility, as well as surgical, oncological, and functional outcomes, have not been proven in large, prospective, and/or randomized studies. Due to the economic and technical differences between Da Vinci and the novel MRS, comparative studies between platforms are essential to fully understand if they can offer an alternative to the standard in renal surgery.

The purpose of this meta-analysis and systematic review is to gather clinical data on robot-assisted partial nephrectomy (RAPN) performed using the currently available MRS and compare it with clinical data from Da Vinci’s experience.

## Evidence acquisition

### Search strategy

The current protocol was registered in the International Prospective Register of Ongoing Systematic Reviews (PROSPERO) on 16th May 2025, and the registration ID is the following: CRD420251041485. The systematic review was carried out in accordance with:

- The EAU Guidelines [7];
- The updated Preferred Reporting Items for Systematic Reviews and Meta-Analyses (PRISMA) recommendations [8];
- Published guidance on performing meta-analysis on prevalence data [9].

The Population-Intervention-Control-Outcome (PICO) framework can be consulted in Supplementary Table 1.

In October 2025, a comprehensive literature search was conducted using the PubMed/Medline, Web of Science, and Scopus databases. There were no language restrictions. Significant studies’ references were manually examined to identify studies of interest using snowballing. The detailed search strategy is available in Supplementary Table 2.

### Study selection, eligibility criteria, and data extraction

Inclusion criteria for studies’ analysis were: At least five adult patients (≥ 18 years old) who had been diagnosed with a renal mass and required surgery; assessed the feasibility of novel MRS in performing RAPN (both transperitoneal and retroperitoneal); evaluated the results on surgical outcomes and oncological outcomes. Theses, case reports, authors’ responses, reviews with or without meta-analysis, and commentaries were not included. The Da Vinci MRS was used as a comparator for RAPN when used in the original papers. Both prospective and retrospective studies published in full text or as conference abstracts were included.

Three independent investigators (F.R., M.M., and F.G.) screened the manuscripts based on titles and abstracts to find reports that were eligible. Pertinent papers received a full-text review and, if suitable, their data were extracted. Two emails were sent to the authors in case any information was missing or unclear. A fourth co-author (B.R.) helped to overcome disagreements.

Three reviewers (F.R., M.M., and F.G.) carried out the data extraction. Discussions were employed to settle disagreements. A data extraction code sheet was used to extract equivalent information systematically. The data extracted were checked twice.

Surgery-related time (docking, console, and operative time), estimated blood loss (EBL), warm ischemia time (WIT), clampless procedures rate, portoperative complications rate according to Clavien Dindo (CD) classification system (relevant postoperative complication defined as CD > 2), length of stay (LOS), and final histopathology according to tumor-nodes-metastasis (TNM) staging were collected as perioperative variables.

Oncological outcomes included the trifecta achievement and the positive surgical margins (PSM) rate. If not otherwise specified in the manuscripts, trifecta was defined as the contemporary presence of negative surgical margins, warm ischemia time < 20 min in case of on-clamp procedure, and no urological intraoperative and postoperative complications [10].

Both surgical and oncological outcomes were considered primary outcomes of the present meta-analysis.

Additional data regarding trocar placement were extracted by both included and non-included studies and shown in Supplementary Table 3.

A Microsoft Excel spreadsheet extracted other characteristics regarding single studies features, patients’ age, gender, body mass index (BMI), preoperative American Association of Anesthesiologists (ASA) score, preoperative imaging tumor diameter, RENAL nephrometry score, preoperative T stage (cT stage) at computerized tomography (CT)/ magnetic resonance (MR), preoperative serum hemoglobin levels (Hb), preoperative serum creatinine levels, and preoperative glomerular filtration rate (GFR) levels.

### Statistical analysis

Stata v19 (Stata Corp, College Station, TX, USA) was used for statistical analyses.

Descriptive analysis of included studies used mean and standard deviation (SD) for quantitative variables, which were calculated using the methods of Wan et al. [11]. The number of occurrences with percentages was estimated for qualitative variables.

Pooled estimates were produced in single-arm and comparative studies, stratified per robotic system. The I^2^ statistic and Cochrane’s Q-test were used to evaluate the heterogeneity of the results of studies. In case of quantitative outcomes, the effect sizes were calculated, and the Sidik-Jonkman method was used for the random-effect model application. In case of qualitative outcomes, raw proportion effect sizes were calculated, and the Sidik-Jonkman method was used for the random-effect model application. A difference between groups analysis was carried out to enlighten any relevant group deviation. Every estimate was presented as a proportion or mean with its 95% confidence interval (CI). The results were visually generated using forest plots.

A quantitative analysis of comparative studies of novel MSR versus the Da Vinci MSR was performed. The I^2^ statistic and Cochrane’s Q-test were used to evaluate the heterogeneity of the results of included studies. Mean differences (MDs) were calculated, and the Sidik-Jonkman method was used when a random-effect model was applied for continuous data. To estimate the log odds ratio (OR) for qualitative variables, the Sidik-Jonkman method in the random-effect model was used. Every estimate was presented as a percentage or mean with its 95% confidence interval (CI). The results were visually generated using Forest plots. In studies that compared more than two novel MRS with Da Vinci MRS, the Da Vinci’s population was divided to reduce biases in meta-analysis results. Due to the low number of studies identified and analyzed, funnel plots were not produced due to low reliability. The p-values were deemed statistically significant for the total effect size below 0.05.

### Risk-of-bias assessment

The non-randomized studies’ quality was evaluated using the Risk of Bias in Non-randomized Studies of Interventions (ROBINS-I) tool [12]. The Risk of Bias for Randomized Studies (RoB2) tool [13] was used to assess the quality of randomized studies. The quality of the eligible studies was independently assessed by two writers, F.R. and M.M. Risk of Bias (RoB) assessment results were displayed using the robvis tool [14]. A third co-author (B.R.) was consulted to settle disagreements.

## Evidence synthesis

### Study characteristics

The PRISMA flow diagram shows the detailed study selection process (Supplementary Fig. 1). Our study strategy identified 5633 manuscripts overall. After intensive screening, 18 eligible papers, published between 2022 and 2025, were identified and selected, analysing data of 631 patients who underwent RAPN using novel MRS totally. Sixteen papers were reported in full-text [15–30] and two were conference abstracts [31, 32]. Two studies [23, 28] analyzed differences between three robotic platforms (Da Vinci MRS, KangDuo MRS, and EGDE MRS), so both KangDuo and EDGE groups were included in our study. One study by Prata et al. [33] was not included in the analysis due to a more recent article published on the same population but was considered fundamental for trocar placement (Supplementary Table 3).

Six studies were conducted retrospectively [15, 17, 18, 20, 31, 32], twelve used a prospective design [16, 19, 21–30]. 6 of them are comparative studies between Da Vinci MRS and novel MRS [15, 19, 23, 28–30]. The novel MRS involved are: Hinotori in two manuscripts [15, 16], Hugo RAS in five manuscripts [17–20, 31], Toumai in three manuscripts [21, 22, 29], Dexter in one manuscript [32], KangDuo in three manuscripts [23, 28, 30], Versius in two manuscripts [24, 25], EDGE in two manuscripts [23, 28], and Carina in two manuscripts [26, 27].

Overall, 18 manuscripts focused on 631 RAPN performed using the novel MRS. As abovementioned, 6 of them were comparative studies between Da Vinci MRS and novel MRS, comparing data with 573 patients who underwent RAPN using Da Vinci MRS.

### Risk of bias

According to RoB-2, the manuscript by Li et al. [30] and by Chen et al. [29] had some concerns regarding RoB. According to ROBINS-I, three studies [15, 16, 19] were assessed to have a low RoB. Six papers [20, 21, 23–25, 28] were assessed to have a moderate RoB. Seven papers [17, 18, 22, 26, 27, 31, 32] were assessed to have a high RoB. Detailed results are shown in Supplementary Fig. 2 and Supplementary Fig. 3.

### Baseline Characteristics

Analyzing demographic data from MRS arm in Table 1a, mean age is 58.4 (SD 6.5) years, mean BMI 25.2 (2.2) kg/m^2^, female patients are 238 (39.8%) on 15 studies [15–26, 28–30], mean tumor diameter is 32 (4.5) mm, mean RENAL nephrometry score is 6.7 (1.1).

**Table 1:** **(a)** Novel MRS baseline characteristics. **(b)** Da Vinci MRS baseline characteristics

Author, year	Country	Study design	Robot used	Patients number	Female (*n*, %)	Age [years] (Mean, SD)	BMI [kg/m^2^] (Mean, SD)	Diabetes (*n*, %)	Hypertension (*n*, %)	Preoperative eGRF [ml/min] (Mean, SD)	Clinical tumor size [mm] (Mean, SD)	RENAL nephrometry score (Mean, SD)	cT1 (*n*, %)	cT2 (*n*, %)	cT3 (*n*, %)
a)															
Motoyama et al. 2023	Japan	R	Hinotori	40	14 (35)	62.5 (10.9)	25.3 (7.8)	13 (32.5)	17 (42.5)			7.8 (1.4)			
Miyamoto et al. 2024	Japan	P	Hinotori	18	8 (44)	69.3 (9.7)	23.7 (3.3)			61.5 (20.1)	38 (20)		18 (100)	0	0
Prata et al. 2025	Italy	R	Hugo	140	51 (36.4)	63.5 (12.4)	26.4 (3.7)	20 (14.3)	71 (50.7)	79.5 (21.6)	33 (18)	6.4 (2.2)	131 (93.5)	9 (6.5)	0
Uleri et al. 2024	Spain	R	Hugo	6							34 (9.5)	8.1 (1.9)			
Bobrowski et al. 2024	Canada	R	Hugo	11	5 (45.5)	58 (18)	28.3 (13.7)	1 (9.09)	4 (36.4)		29 (15)				
Garcia Rojo et al. 2024	Spain	P	Hugo	25	9 (36)	61.5 (9.7)	27.9 (3.89)	3 (12)	8 (32)		35 (21.3)	5.8 (2.8)			
Gallioli et al. 2023	Spain	R	Hugo	10	4 (40)	68 (12)				87.1 (20.6)	30 (12.9)				
Tan et al. 2024	China	P	Toumai	5	1 (20)	44.4 (39-61) [Mean, Range]	27.4 (21.2-31-3) [Mean, Range]			109.9 (95.7-118.7) [Mean, Range]					
Pokhrel et al. 2025a	China	P	Toumai	11	4 (36.4)	55.7 (12)	25.1 (3.7)			98.5 (12.6)					
Chen et al. 2025	China	P	Toumai	26	16 (71.5)	62.1 (6.8)	23.5 (2.4)								
Fontanier et al. 2024	France	R	Dexter	20		66.6 (12.8)				78.8 (24.6)					
Li et al., 2022	China	P	KangDuo	49	22 (44.9)	54.4 (10.3)	25.8 (2.8)			93 (16.3)			49 (100)	0	0
Gupta et al., 2025	China	P	KangDuo	40	14 (35)	56.1 (11.2)	25.9 (4.2)	11 (27.5)	8 (20)	83.3 (21.6)					
Gupta et al., 2025	China	P	EDGE	45	22 (48.9)	55.7 (11.7)	24.9 (2.4)	8 (17.8)	12 (26.7)	77.2 (13.3)					
Abdelhakim et al. 2024	Egypt	P	Versius	30	12 (40)	51.3 (13)	28 (3.7)	6 (20)	8 (26.7)	85.4 (14.6)	33.6 (23.4)				
Meneghetti et al. 2024	Italy	P	Versius	13	7 (53.8)	62.5 (11.6)				0.9 (0.2)	36 (24.1)				
Pokhrel et al. 2025b	China	P	Carina	10	2 (20)	57.4 (19.4)	24.4 (3)			89.4 (25.7)					
Wang et al. 2025	China	P	Carina	7		48.3 (8.7)	24.2 (3)				23 (14)	5.8 (2.2)			
Guo et al. 2025	China	P	KangDuo	79	35 (44.3)	55.8 (10.1)	20.7 (3)	12 (15.2)	18 (22.8)				76 (96.2)	3 (3.8)	0
Guo et al. 2025	China	P	EDGE	46	12 (26.1)	56.2 (9)	21.5 (3.1)	5 (10.9)	12 (26.1)				45 (97.8)	1 (2.2)	0
**b)**															
Motoyama et al. 2023	Japan	R	Da Vinci	303	96 (31.7)	64.4 (12.5)	24.3 (6)	58 (19.1)	124 (40.9)			7 (1.2)			
Garcia Rojo et al. 2024	Spain	P	Da Vinci	25	6 (24)	63.6 (9.3)	26.5 (4.1)	4 (16)	10 (40)		33 (11.7)	5.6 (2.1)			
Chen et al. 2025	China	P	Da Vinci	25	13 (52)	60.1 (13.1)	23.4 (2.7)								
Li et al. 2022	China	P	Da Vinci	50	15 (30)	52.1 (12.4)	25.6 (3.3)			93 (16.3)			50 (100)	0	0
Gupta et al. 2025	China	P	Da Vinci	85	28 (32.9)	55.3 (10.5)	26.2 (4.9)	15 (17.6)	22 (25.9)	79.5 (16.7)					
Guo et al. 2025	China	P	Da Vinci	85	39 (45.9)	57 (8.3)	21.7 (3.3)	9 (10.6)	19 (22.4)				83 (97.6)	2 (2.4)	0

MRS, multiport robotic system; P, prospectical; R, retrospective; BMI, body mass index; eGFR, estimated glomerular filtration rate; SD, standard deviation

Demographic data of Da Vinci arm can be found in Table 1b. In the Da Vinci arm mean age is 58.8 (11.7) years, mean BMI is 24.6 (4.1) kg/m^2^, female patients are 197 (34.4%) on 6 studies [15, 19, 23, 28–30].

### Clinical outcomes

In Table 2a, novel MRS arm: mean docking time is 7.4 (3.2) minutes, mean console time is 107.2 (31) minutes, mean operative time is 155.6 (32.9) minutes, mean EBL is 112.6 (63.7) mL, mean WIT is 18.2 (6.7) minutes, and mean LOS is 4.3 (2.3) days. Overall, 120 (27.2%) procedures were performed clampless on 13 studies [15, 17–23, 25, 29–32], and 15 (2.4%) postoperative complications CD > 2 occurred on 17 studies [15–26, 28–32]. Only 3 (0.6%) intraoperative conversions to open or laparoscopic surgery occurred on 13 studies [15, 16, 18–20, 22–26, 28–30].

**Table 2:** **(a)** Novel MRS perioperative and surgical outcomes. **(b)** Da Vinci MRS perioperative and surgical outcomes

References	Robot used	Patients number	Docking time [min] (Mean, SD)	Console time [min] (Mean, SD)	Operative time [min] (Mean, SD)	Estimated blood loss [mL] (Mean, SD)	Warm ischemia time [min] (Mean, SD)	Clampless procedures (n, %)	Major postoperative complications [CD>2] (n, %)	Conversion to open/LPS (n, %)	Length of stay [days] (Mean, SD)
**a)**											
Motoyama et al. 2023	Hinotori	40		106 (32)	173.4 (37.6)	59.4 (72.1)	12.1 (3.5)	0	0	0	7.5 (4.4)
Miyamoto et al. 2024	Hinotori	18		150.7 (53.1)	241.2 (68.4)	131.7 (104.6)	20.6 (5.6)		0	0	
Prata et al. 2025	Hugo	140	5.4 (2.2)	86.9 (67)	150.2 (81.6)	185.1 (149.8)	16.4 (6.7)	90 (64.3)	7 (5)		3.4 (0.7)
Uleri et al. 2024	Hugo	6	6 (1)		177.7 (38.1)	88.5 (28.6)	16.4 (4.8)	0	0		3.4 (1)
Bobrowski et al. 2024	Hugo	11	3.9 (1.7)	93 (21.4)	165.6 (34.1)	179 (63.6)	18.9 (7.1)	0	0	0	2 (1)
Garcia Rojo et al. 2024	Hugo	25	20.1 (0.3)	103.5 (46.5)		142.4 (85.3)	9.9 (11.9)	14 (56)	0	0	2.4 (0.7)
Gallioli et al. 2023	Hugo	10	11 (4.3)	141.7 (32.7)		88.2 (21.5)	12.3 (9.4)	1 (10)	1 (10)	1 (10)	4.4 (2.6)
Tan et al. 2024	Toumai	5	12.8 (7)		175 (85-210) [Mean, Range]		34.6 (20-50) [Mean, Range]	0	0		9.4 (6-12) [Mean, Rnage]
Pokhrel et al. 2025a	Toumai	11	9.4 (4.2)		101.4 (36.4)	54.3 (12)	10.1 (6.2)	1 (9.1)	0	0	
Chen et al. 2025	Toumai	26	4.1 (0.9)		147 (17.7)		22.4 (3)	0	0	0	
Fontanier et al. 2024	Dexter	20			175.6 (47.1)	275.7 (251.3)	25.1 (12.9)	4 (20)	4 (20)		2.7 (1.6)
Li et al. 2022	KangDuo	49	0.2 (1.7)	69.6 (30.9)		35.8 (30.5)	18.4 (5.5)	3 (6.1)	0	0	4.4 (1.6)
Gupta et al. 2025	KangDuo	40	0.2 (0.1)		129 (38.2)	75.5 (42)		0	0	0	
Gupta et al. 2025	EDGE	45	4.3 (1.4)		145.2 (52.9)	61.6 (30.6)		0	0	0	
Abdelhakim et al. 2024	Versius	30	9.2 (0.9)	149 (14.3)	177.2 (29.5)	154.33 (67.2)	26.7 (3.7)		3 (10)	0	2.4 (0.5)
Meneghetti et al. 2024	Versius	13		73.5 (11.6)	105 (8.3)	181.8 (124.6)	10 (1.7)	7 (53.8)	0	2 (13.3)	3.6 (0.8)
Pokhrel et al. 2025b	Carina	10	6.7 (4.1)		120.3 (46.4)	55.8 (34.2)	12.6 (7)		0	0	
Wang et al. 2025	Carina	7	8.9 (1.6)	62.6 (24.9)	146 (38)	47.2 (7.6)	23.9 (6.7)				6.4 (1.5)
Guo et al. 2025	KangDuo	79	4.4 (1.3)	126.4 (43.3)	162.4 (45.3)	114 (79.1)	18.3 (4.2)		0	0	
Guo et al. 2025	EDGE	46	4.6 (1.1)	122.4 (35.4)	153.7 (48.5)	96.9 (26.7)	18.3 (4.1)		0	0	
**b)**											
Motoyama et al. 2023	Da Vinci	303		107 (26.5)	175.3 (60.8)	156.9 (731.2)	13.6 (7)	55 (18.5)	9 (3)	2 (0.7)	8.5 (4.7)
Garcia Rojo et al. 2024	Da Vinci	25	12.6 (3)	102.3 (45.1)		233.8 (265.8)	14.4 (15.6)	11 (44)	0	0	2.2 (0.6)
Chen et al. 2025	Da Vinci	25	3.1 (0.6)		127 (16.2)		21.9 (3.6)	0	0	0	
Li et al. 2022	Da Vinci	50	3.4 (1.3)	61.5 (19.7)		33.5 (22.9)			0		4.4 (0.9)
Gupta et al. 2025	Da Vinci	85			161.4 (36.9)	79.5 (42.9)			0	0	
Guo et al. 2025	Da Vinci	85	3 (1)	99.3 (31.1)		79.8 (31.3)	17.3 (4.4)		0		

MRS, multiport robotic system; CD, Clavien Dindo; LPS, laparoscopy; SD, standard deviation

In Table 2b, Da Vinci MRS arm: mean docking time is 5.5 (1.7) minutes, mean console time is 92.5 (3.5) minutes, mean operative time is 154.6 (21.1) minutes, mean EBL is 116.7 (43.2) mL, mean WIT is 16.8 (1.8) minutes, mean LOS is 5 (2.6) days, 9 (1.6%) postoperative major complications (CD > 2) occur on 6 studies [15, 19, 23, 28–30], 66 (18.7%) procedures are performed clampless on 3 studies [15, 19, 29], and 2 (0.5%) cases are converted to open/laparoscopic surgery on 4 studies [15, 19, 23, 29].

Pooled estimates are produced and stratified according to novel MRS adopted. Pooled effect sizes are shown in Supplementary Figs. 4–10. Pooled mean docking time is 6.55 (95% CI 4.03–9.07; I^2^ 98.23%; subgroup effect *p* < 0.01) minutes. Pooled mean console time is 103.96 (95% CI 82.9–125; I^2^ 44.5%; subgroup effect *p* = 0.64) minutes. Pooled mean operative time is 145.04 (95% CI 125.7-164.3; I^2^ 30.7%; subgroup effect *p* = 0.91) minutes. Pooled mean EBL is 78.1 (95% CI 52.7-103.5; I^2^ 61.3%; subgroup effect *p* = 0.14) mL. Pooled mean WIT is 16.64 (95% CI 13.1–20.2; I^2^ 79.97%; subgroup effect *p* = 0.97) minutes. Pooled major postoperative complication (CD > 2) rate is 0.02 (95% CI 0.01–0.04; I^2^ 58.1%; subgroup effect *p* = 0.19). Pooled LOS is 3.74 (95% CI 2.51–4.97; I^2^ 75.5%; subgroup effect *p* = 0.01) days.

### Oncological outcomes

Table 3a and Table 3b provide analyses of postoperative, oncological, and functional outcomes, respectively in novel MRS and Da Vinci MRS arms. In novel MRS group, malignant tumors at pathology are 352 (83%) on 11 studies [15–24, 30] and 265 (62.5%) are clear cell carcinomas at pathology, 290 (87.9%) tumors are pT1 at histology on 9 studies [16–21, 23, 24, 31]. PSM are found in 13 (2.2%) patients on 16 studies [15–26, 28–31]. Trifecta is achieved in 97 (91.5%) cases on 4 studies [15, 19, 22, 24].

**Table 3:** **a)** Novel MRS Oncological outcomes.**b)** Da Vinci MRS Oncological outcomes

References	Robot used	Patients number	Malignant tumor rate (n, %)	pT1 rate (n, %)	Positive Surgical Margins (n, %)	Trifecta achievement rate (n, %)	Readmission rate (n, %)
**a)**							
Motoyama et al. 2023	Hinotori	40	39 (97.5)		0	40 (100)	
Miyamoto et al. 2024	Hinotori	18	17 (94.4)	13 (77)	0		
Prata et al. 2025	Hugo	140	105 (75)	123 (87.9)	10 (7.1)		
Uleri et al. 2024	Hugo	6		6 (100)	0		
Bobrowski et al. 2024	Hugo	11	11 (100)	11 (100)	0		0
Garcia Rojo et al. 2024	Hugo	25	19 (76)	23 (92)	2 (8)	21 (84)	
Gallioli et al. 2023	Hugo	10	5 (50)	4 (40)	0		
Tan et al. 2024	Toumai	5	5 (100)	5 (100)	0		
Pokhrel et al. 2025a	Toumai	11	11 (100)		0	11 (100)	0
Chen et al. 2025	Toumai	26			0		
Fontanier et al. 2024	Dexter	20					
Li et al. 2022	KangDuo	49	38 (77.5)		0		
Gupta et al., 2025	KangDuo	40	33 (82.5)	39 (97.5)	0		
Gupta et al. 2025	EDGE	45	39 (86.7)	44 (97.8)	0		
Abdelhakim et al. 2024	Versius	30	30 (100)	22 (73.4)	0	25 (83.3)	
Meneghetti et al. 2024	Versius	13			1 (7.7)		0
Pokhrel et al. 2025b	Carina	10			0		
Wang et al. 2025	Carina	7					
Guo et al. 2025	KangDuo	79			0		
Guo et al. 2025	EDGE	46			0		
**b)**							
Motoyama et al. 2023	Da Vinci	303	248 (81.8)		2 (0.7)	289 (95.4)	
Garcia Rojo et al. 2024	Da Vinci	25	22 (88)	24 (96)	1 (4)	22 (88)	
Chen et al. 2025	Da Vinci	25			0		
Li et al. 2022	Da Vinci	50	40 (80)		0		
Gupta et al. 2025	Da Vinci	85	81 (95.3)	74 (87.1)			
Guo et al. 2025	Da Vinci	85			0		

MRS, multiport robotic system

In the Da Vinci arm malignant tumors at pathology are 391 (84.4%) on 4 studies [15, 19, 23, 30], PSM are found in 3 (0.6%) patients on 5 studies [15, 19, 28–30], trifecta is achieved in 311 (94.8%) cases on 2 studies [15, 19].

As shown in Supplementary Fig. 11, pooled PSM rate is 0.02 (95% CI 0.01–0.03; I^2^ 32.3%; subgroup effect 0 = 0.13).

### Statistical analysis between novel MRS and Da Vinci MRS

**Fig. 1:**
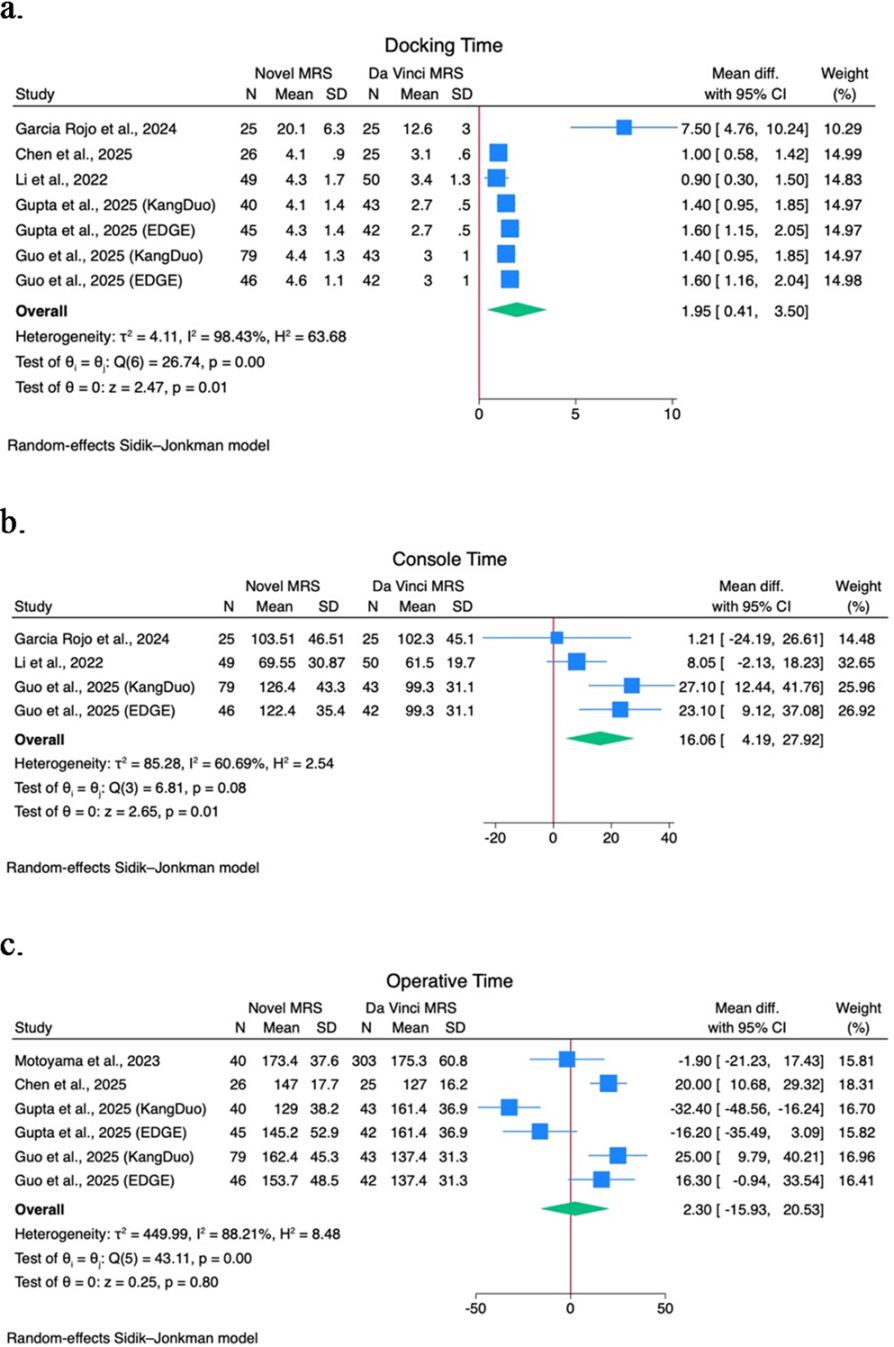
(**a**) Docking time forest plot; (**b**) Console time forest plot; (**c**) Operative time forest plot

**Fig. 2:**
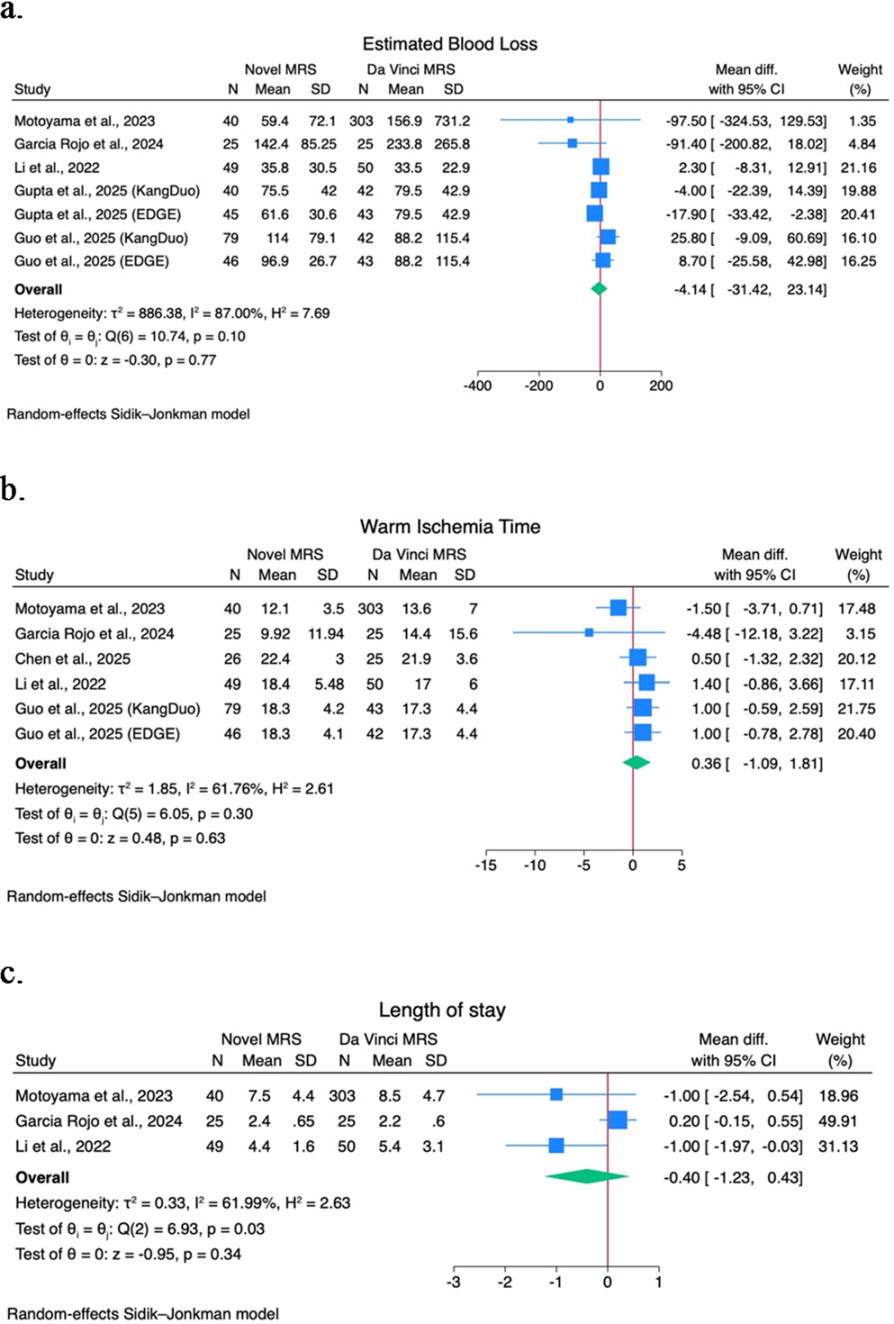
(**a**) Estimated blood loss forest plot; (**b**) Warm ischemia time forest plot; (**c**) Length of stay forest plot

**Fig. 3:**
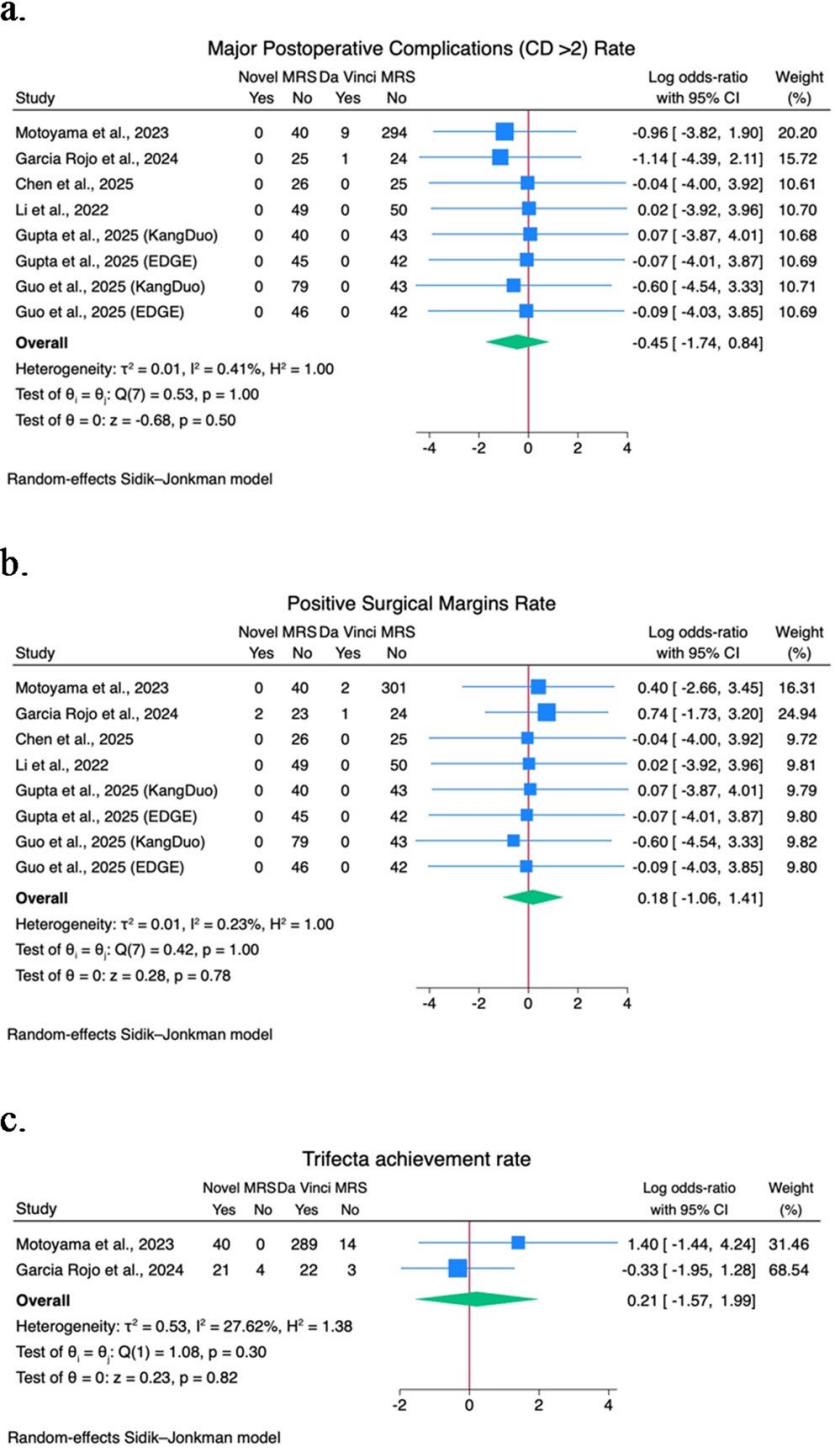
**a**) Major postoperative complication (CD > 2) rate forest plot; **b**) Positive surgical margins forest plot; **c**) Trifecta achievement rate forest plot

A quantitative analysis of comparative studies of novel MRS versus the Da Vinci MRS was performed on docking time, console time, operative time, EBL, WIT, LOS, major postoperative complications (CD > 2) rate, PSM rate, and trifecta achievement rate. Forest plots are shown in Figs. 1 and 2, and Fig. 3.

A statistically significant mean difference of 1.95 (95% CI 0.41–3.5; I^2^ 98.4%; *p* = 0.01) minutes is shown in docking time among 5 studies [19, 23, 28–30]. A statistically significant mean difference of 16.06 (95% CI 4.2–27.9; I^2^ 60.7%; *p* = 0.01) minutes is shown in console time among 3 studies [19, 28, 30].

No statistically significant mean differences are shown in terms of operative time (2.3; 95% CI -15.9–20.5; I^2^ 88.2; *p* = 0.80) among 4 studies [15, 23, 28, 29], EBL (-4.14; 95% CI -31.4–23.14; I^2^ 87%; *p* = 0.77) among 5 studies [15, 19, 23, 28, 30], WIT (0.36; 95% CI -1.09–1.8; I^2^ 61.8%; *p* = 0.6) among 5 studies [15, 19, 28–30], LOS (-0.4; 95% CI -1.2–0.4; I^2^ 61.99; *p* = 0.34) among 3 studies [15, 19, 30].

No statistically significant differences are shown in major postoperative complications (CD > 2) rate (logOR − 0.45; 95% CI -1.74–0.84; I^2^ 0.41%; *p* = 0.5) among 6 studies [15, 19, 23, 28–30], PSM rate (logOR 0.18; 95% CI -1.06–1.41; I^2^ 0.23%; *p* = 0.78) among 6 studies [15, 19, 23, 28–30], and trifecta achievement (logOR 0.21; 95% CI -1.57–1.99; I^2^ 27.6%; *p* = 0.82) among 2 studies [15, 19].

## Discussion

The present systematic review and meta-analysis compares surgical and early oncological outcomes of patients undergoing RAPN with the novel MRS and Da Vinci surgical systems. Our topic has been faced in a previous meta-analysis, showing encouraging results and important points of view on novel MRS adoption in renal surgery [34].

However, our manuscript reinforces these findings from comparative studies with the Da Vinci system, which is considered the reference standard due to its 20-years use, adding the more recent studies on RAPN approached novel MRS. The current manuscript confirms that several novel MRS —Hinotori, Hugo RAS, KangDuo, Toumai, Versius, EDGE, Carina, and Dexter— have now been applied to RAPN with acceptable peri- and post-operative outcomes. However, their penetration into this indication remains notably lower than what has been observed for radical prostatectomy. For instance, Reitano et al. [34], documented a robust body of literature on new robotic platforms used in robot-assisted radical prostatectomy (RARP), with numerous descriptive (26 single-arm studies) and comparative studies (14 comparative studies) supporting safety, feasibility, and functional outcomes across different systems. In contrast, the present review identified only 18 studies focusing on RAPN, with 6 of them being comparative, underscoring the disparity in diffusion and documentation between these two urological procedures. While this likely reflects the current geographical distribution of the robotic surgery market, it may also indicate a more cautious and gradual adoption of novel robotic platforms for partial nephrectomy across European countries.

Acknowledging the heterogeneity and pioneering nature of most included series, our synthesis —comprising a total of 1204 patients— demonstrates that novel robotic systems yield perioperative outcomes comparable to those reported with Da Vinci–based platforms. Our results indicate that RAPN conducted with the various robotic platforms produce similar outcomes. The implementation of the novel MRS in clinical settings for renal surgery is both safe and practical. Urological robotic surgeries utilizing the innovative MRS should be carried out in larger robotic centers, reinforcing existing data in the literature without increasing surgical, oncological, or functional risks associated with inexperience and surgeons who are still on their learning curve.

Our results showed a statistically significant difference in docking and console time between Da Vinci and novel MRS, as can be seen in other meta-analyses [35]. Besides several similarities between these platforms, the initial approach to new mechanics and interfaces may play a role in the time spent moving the single-boom or the multi-cart and the time spent by the surgeon at the console, especially during the first cases. Although these findings were statistically significant, no clinically significant differences were noticed by the authors in all the studies analyzed.

No other statistically significant differences emerged between the platforms. Operative time, EBL, WIT, LOS, and CD > 2 complication did not show any statistically significant difference, so we can conclude that intraoperative safety and feasibility is confirmed. Novel MRS are not more time-consuming than Da Vinci MRS, ensuring technical quality and a smooth transition between platforms for multi-platform users since first perfomances.

The non-statistically significant differences in terms of PSM rate and trifecta achievement underscore the absence of clinically significant learning curve in the transition period, moving from Da Vinci system to the novel robotic platform. It can be seen also in other urologic surgical procedures, in accordance with the current literature [36].

Supplementary Table 3 shows off the variability that can be found in trocar placement when performing a RAPN. Fourth arm usage is not mandatory in the present urological procedure, but it could be useful. A recent study evidenced the role of the fourth arm during RAPN, concluding that in tumors located in the inner side of the kidney the fourth arm could not be game-changing, so its usage should be evaluated and considered for each tumor location [37].

Almost every author in the present paper considers important the placement of a 12 mm AirSeal trocar in both novel MRS and Da Vinci MRS, due to its higher safety in gas supply, as can be seen in a recent meta-analysis [38].

A thorough analysis of the technical characteristics of all the platforms studied shows various strengths and weaknesses related to their specific features. Nearly every novel MRS aimed to provide distinct potential benefits regarding ergonomics, communication, sustainability, and long-distance training.

The Hugo™ RAS system introduces distinctive elements such as an open console design, pistol-like hand controllers, sing cart docking model, and advanced visualization tools [35, 39]. However, the potential risks of external distractions associated with an open console design, which could affect surgical performance, should be acknowledged.

Toumai MT-1000 surgical platform includes several features, such as four robotic arms, a stereoscopic view providing a 3-dimensions (3D) experience for surgeons, Picture-in-Picture availability, high image resolution, a low master operating force (≤ 0.1 N), 5G Remote Operation with a master-slave delay of ≤ 70 ms, which becomes fundamental in the telesurgery field [40]. This revolutionary patent gives to Toumai the opportunity to unlock the remote training worldwide. However, some ethical aspects must be faced [41, 42].

Dexter robotic system, thanks to its two robotic carts and an endoscope arm, perfectly replicates laparoscopic setting, minimizing transition from laparoscopic to robotic surgery [43].

KangDuo surgical system and Versius surgical system provide an open console, reducing neck fatigue experienced with Da Vinci [44]. The former is equipped with 3 robotic arms, the latter with 4 robotic arms. Both can use the commercially available three-dimensional endoscopy systems (e.g. Olympus and Storz).

The Hinotori robotic platform represents a paradigm shift in robotic surgery through its ultra-slim 4-axis arms, specifically engineered to eliminate the “arm-to-arm” collisions. This compact architecture maximizes bedside assistant accessibility, enhancing intraoperative efficiency, providing larger space in a clean field for table assistants and avoiding collision between robotic arms outside from the patient’s body [45].

The Carina robotic system optimizes operating room space thanks to its ultra-compact structure and allows a flexible positioning tailored to specific surgical needs. The haptic feedback technology provides a tactile sense of tissue resistance, often missing in traditional platforms. The open-design console enhances team communication and reduces surgeon fatigue through superior ergonomics [27].

However, robotic platforms’ reliability should be investigated. A recurring limitation across the reviewed studies is the sparse reporting of device-specific malfunctions or technical failures. Considering the complex nature of partial nephrectomy and the reliance on robotic platforms, standardized reporting on intraoperative technical issues is critical to fully assess feasibility and safety. Recently, a pivotal meta-analysis by Buffi et al. [46] faced the theme of technical robotic failures, underscoring the trustworthiness of the Da Vinci robotic platform, confirming its role as the robotic standard. Comparative studies could be important to assess differences between platforms in terms of technical failures and future investigations should explicitly address technological breakdowns, instrument issues, and intraoperative interruptions, to inform both clinical practice and device improvement.

Moreover, comprehensive training programs and the standardization of protocols are essential to fully maximize the benefits of the new robotic systems and to improve its effect on operative times, as highlighted in a paper by Olsen et al. [47] on the impact of Hugo’s features on an experienced scrub nurse team. The learning curve and work flow an experienced robotic nurse team transitioning from Da Vinci to the Hugo RAS system were examined. No learning curve plateau was reached. Even after 30 RARP, indicating long time to gain expertise in the scrab part. However, the work pattern seemed to arrive at a plateau after 20 procedures, gaining proficiency in multitasking between the scrub nurse and the circulating nurse.

An important consideration is the potential cost advantage of the novel platforms. It can be seen with Medtronic’s Hugo RAS [18, 48]. A Da Vinci treatment costs €2,246.31, while a Hugo-RAS procedure costs €1,995, according to Sighinolfi et al. This means that RARP using the HugoTM RAS System in a local setting saves 11% of the total cost. This per-operation cost, when combined with clinical data, results in an overall index hospitalization cost of €6,775.51 for the Da Vinci procedure and €6,637.15 for the Hugo RAS surgery. In renal surgery, this type of analysis should be performed, trying to put in evidence an economical advantage in novel MRS use. Further cost-effectiveness studies are necessary to validate this assumption and provide a comprehensive understanding of the economic implications of adopting the novel MRS in a robotic-naive setting.

### Study limitations

Despite the systematic approach, the small number of direct comparative studies (only 6 with 5 different robotic systems) precluded statistically strong meta-analytic synthesis. The lack of comparative studies may reflect logistical challenges, availability, or early diffusion preferences for partial nephrectomy across institutions—but again, this underlines the need for more balanced, inclusive comparative research across different configurations.

Several limitations need to be acknowledged. The small sample sizes of the included studies may not identify or warp differences in terms of outcomes between different robotic systems, so larger studies with diverse populations are needed.

Furthermore, the current evidence represents initial findings in the comparison between the two robotic populations. Future studies could give new information, as surgeons would gain more skills and would reach robotic proficiency on novel MRS. Prospective and randomized studies with larger cohorts are needed to validate these findings and assess long-term oncological and functional outcomes.

High and significant heterogeneity can be found in the analysis of some outcomes, which may be attributed to the inclusion of various types of study designs. This variability could potentially impact the reliability of the meta-analysis. A potentially impactful publication bias and small study bias may be found, reducing statistical strength of our findings, but the low reliability of funnel plots could not help us in identification of more biased studies. Larger comparative studies are necessary to conclusively define the non-inferiority of novel MRS when compared with Da Vinci MRS.

Moreover, ongoing advancements in robotic technology may introduce additional considerations and complexities that warrant continued evaluation to ensure that the findings remain relevant as the technology evolves.

### Take-home messages

1. RAPN performed with the novel MRS have shown perioperative, oncological, and functional outcomes similar to those achieved with the well-known Da Vinci system.
2. Docking and console times are longer in novel platforms when compared with Da Vinci MRS, suggesting the need for pre-clinical training programs.
3. Surgeons do not experience an oncologically-significant learning curve with the novel MRS, indicating the standardization of surgical technique as fundamental for robotic transition.
4. The novel MRS offer ergonomics and technical advantages like the Da Vinci system, but their safety and reliability should be investigated deeper.

## Conclusion

The present systematic review and meta-analysis provide important insights regarding outcomes of RAPN performed with the novel MRS when compared with the Da Vinci surgical system. The docking times and console times were significantly shorter for the Da Vinci system. However, other compared clinical parameters were not either statistically or clinically significantly different, underscoring the feasibility and safety of RAPN performed using novel MRS. According to these findings, further research and clinical experience need to assess the role of innovative robotic platforms in urological surgery from now on. In the future, data from prospective and randomized studies on novel MRS with larger sample sizes may validate these findings and provide more concrete guidance for surgical decision-making.

## Supplementary Information

Below is the link to the electronic supplementary material.

Supplementary Material 1

## Data Availability

The data presented in this study are available on request from the corresponding author.
