# Supplementary material for: Novel multiport robotic systems versus da vinci multiport robotic system in robot-assisted partial nephrectomy: a systematic review and meta-analysis of surgical and oncological outcomes
Source: J Robot Surg. 2026 Mar 5;20(1):314. doi: 10.1007/s11701-026-03277-w (PMC12960315; doi:10.1007/s11701-026-03277-w)

| ***Supplementary Table 1.*** *PICOS framework* |  |
| --- | --- |
| **Population** | Adult patients [aged ≥ 18 years] diagnosed with kidney cancer |
| **Intervention** | Patients who underwent robotic-assisted partial nephrectomy with the novel robotic multiport systems |
| **Comparison** | Adult patients who underwent robotic-assisted partial nephrectomy with Da Vinci robotic surgical system |
| **Outcomes** | surgical, oncological, and functional outcomes |

| ***Supplementary Table 2.*** *Searching strategy* |
| --- |
| **Pubmed: 1722 results** |
| ((“HUGOTM”[All Fields] OR “HUGO”[All Fields] OR “HUGO RAS”[All Fields] OR “HUGO TM”[All Fields] OR “HUGORAS”[All Fields] OR “HUGO-RAS”[All Fields] OR “HUGO-TM”[All Fields]) OR (“Versius”[All Fields] OR “CMR”[All Fields] OR “Versius CMR”[All Fields]) OR (“KangDuo”[All Fields] OR “Kang Duo”[All Fields] OR “Kang-Duo”[All Fields]) OR (“Senhance”[All Fields]) OR (“Avatera”[All Fields]) OR (“Edge”[All Fields]) OR (“Carina”[All Fields]) OR (“Toumai”[All Fields]) OR (“Hinotori”[All Fields]) OR (“Dexter”[All Fields]) OR (“Revo-i”[All Fields] OR “Revoi”[All Fields] OR “ev oi”[All Fields])) AND ((“robotic-assisted”[All Fields] OR “robot-assisted”[All Fields] OR “robot assisted”[All Fields] OR “robotic assisted”[All Fields] OR (“robotic”[All Fields] AND “surgical”[All Fields] AND “procedures”[All Fields]) OR “robotic surgical procedures”[All Fields] OR (“robotic”[All Fields] AND “surgery”[All Fields]) OR “robotic surgery”[All Fields] OR “robot*”[All Fields] OR “surg*”[All Fields]) AND (“nephrectomy”[All Fields] OR “nephrectomies”[All Fields] OR “heminephrectomy”[All Fields] OR “heminephrectomies”[All Fields] OR “partial”[All Fields] OR “partials”[All Fields] OR “kidney cancer”[All Fields] OR “kidney”[All Fields] OR “renal”[All Fields] OR “kidney neoplasms”[All Fields] OR “kidney neoplasm”[All Fields])) |
| **Web of Science: 1302 results** |
| TS=( ((“HUGOTM” OR “HUGO” OR “HUGO RAS” OR “HUGO TM” OR “HUGORAS” OR “HUGO-RAS” OR “HUGO-TM”) OR (“Versius” OR “CMR” OR “Versius CMR”) OR (“KangDuo” OR “Kang Duo” OR “Kang-Duo”) OR (“Senhance”) OR (“Avatera”) OR (“Toumai”) OR (“Carina”) OR (“Edge” OR “EDGE”) OR (“Hinotori”) OR (“Dexter”) OR (“Revo-i” OR “Revoi” OR “ev oi”)) AND ((“robotic-assisted” OR “robot-assisted” OR “robot assisted” OR “robotic assisted” OR (“robotic” AND “surgical” AND “procedures”) OR “robotic surgical procedures” OR (“robotic” AND “surgery”) OR “robotic surgery” OR “robot*” OR “surg*”) AND (“nephrectomy” OR “nephrectomies” OR “heminephrectomy” OR “heminephrectomies” OR “partial” OR “partials” OR “kidney cancer” OR “kidney” OR “renal” OR “kidney neoplasms” OR “kidney neoplasm”)) ) |
| **Scopus: 2609 results** |
| ( ( TITLE-ABS-KEY ( “HUGOTM” ) OR TITLE-ABS-KEY ( “HUGO” ) OR TITLE-ABS-KEY ( “HUGO RAS” ) OR TITLE-ABS-KEY ( “HUGO TM” ) OR TITLE-ABS-KEY ( “HUGORAS” ) OR TITLE-ABS-KEY ( “HUGO-RAS” ) OR TITLE-ABS-KEY ( “HUGO-TM” ) ) OR ( TITLE-ABS-KEY ( “Versius” ) OR TITLE-ABS-KEY ( “CMR” ) OR TITLE-ABS-KEY ( “Versius CMR” ) ) OR ( TITLE-ABS-KEY ( “KangDuo” ) OR TITLE-ABS-KEY ( “Kang Duo” ) OR TITLE-ABS-KEY ( “Kang-Duo” ) ) OR TITLE-ABS-KEY ( “Senhance” ) OR TITLE-ABS-KEY ( “Avatera” ) OR TITLE-ABS-KEY ( “Toumai” ) OR TITLE-ABS-KEY (“Carina”) OR TITLE-ABS-KEY (“Edge”) OR TITLE-ABS-KEY ( “Hinotori” ) ) AND ( ( TITLE-ABS-KEY ( “robotic-assisted” ) OR TITLE-ABS-KEY ( “robot-assisted” ) OR TITLE-ABS-KEY ( “robot assisted” ) OR TITLE-ABS-KEY ( “robotic assisted” ) OR ( TITLE-ABS-KEY ( “robotic” ) AND TITLE-ABS-KEY ( “surgical” ) AND TITLE-ABS-KEY ( “procedures” ) ) OR TITLE-ABS-KEY ( “robotic surgical procedures” ) OR ( TITLE-ABS-KEY ( “robotic” ) AND TITLE-ABS-KEY ( “surgery” ) ) OR TITLE-ABS-KEY ( “robotic surgery” ) OR TITLE-ABS-KEY ( “robot*” ) OR TITLE-ABS-KEY ( “surg*” ) ) AND ( TITLE-ABS-KEY ( “nephrectomy” ) OR TITLE-ABS-KEY ( “nephrectomies” ) OR TITLE-ABS-KEY ( “heminephrectomy” ) OR TITLE-ABS-KEY ( “heminephrectomies” ) OR TITLE-ABS-KEY ( “partial” ) OR TITLE-ABS-KEY ( “partials” ) OR TITLE-ABS-KEY ( “kidney cancer” ) OR TITLE-ABS-KEY ( “kidney” ) OR TITLE-ABS-KEY ( “renal” ) OR TITLE-ABS-KEY ( “kidney neoplasms” ) OR TITLE-ABS-KEY ( “kidney neoplasm” ) ) ) |

| ***Supplementary Table 3.*** *Trocar placement* | | | | | | | | | | | | | |
| --- | --- | --- | --- | --- | --- | --- | --- | --- | --- | --- | --- | --- | --- |
|  |  | **Transperitoneal trocar placement** | | | | | | **Retroperitoneal trocar placement** | | | | | |
| Author, year | Robot used | Optic trocar | Right hand instrument | Left hand instrument | Forth arm instrument | Bed assistant 1 | Bed assistant 2 | Optic trocar | Right hand instrument | Left hand instrument | Forth arm instrument | Bed assistant 1 | Bed assistant 2 |
| Prata et al., 2023 | Hugo | 11 mm trocar, pararectal line, 14 cm far below xifopubic line | 8 mm trocar, 8 cm laterally to camera port, 2 cm from bone prominencies | 8 mm trocar, 8 cm laterally to camera port, 2 cm from bone prominencies |  | 12 mm trocar, 8 cm medially from robotic ports | 12 mm trocar, 8 cm medially from robotic ports |  |  |  |  |  |  |
| Uleri et al., 2024 | Hugo |  |  |  |  |  |  | 11 mm trocar, 2 cm under the tip of the 12th rib | 8 mm trocar, posterior axillary line | 8 mm trocar, following an inline configuration | 8 mm trocar, following an inline configuration |  |  |
| Bobrowski et al., 2024 | Hugo | 11 mm trocar | 8 mm trocar, at the pararectus line, 8-10 cm from the endoscope port | 8 mm trocar, at the pararectus line, 8-10 cm from the endoscope port |  | 12 mm trocar, near the umbilicus | 5 mm trocar for liver retraction in right RAPN. Additionally, another 5 mm trocar was placed for bed assistant |  |  |  |  |  |  |
| Garcia Rojo et al., 2024 | Hugo | 11 mm trocar, 5 cm below the rib margin on the mid-clavicular line | 8 mm trocar, on the pararectal line | 8 mm trocar, on the pararectal line | 8 mm trocar, 2 cm above the mid-clavicular line | 12 mm trocar | Additional 5 mm trocar for liver retraction |  |  |  |  |  |  |
| Gallioli et al., 2023 | Hugo | 11 mm trocar, 5 cm under the ribs' margin on the mid-clavicular line | 8 mm trocar, on the pararectal line, maintaining 8 cm from the endoscope trocar | 8 mm trocar, on the pararectal line, maintaining 8 cm from the endoscope trocar | 8 mm trocar, 2 cm above the mid-clavicular line, 8 cm far from the right-hand trocar | 12 mm trocar, under the endoscope trocar, 2 cm far from other trocars and bony prominences | 5 mm trocar, cranially to the right arm trocar for liver retraction |  |  |  |  |  |  |
| Li et al., 2022 | KangDuo | 12 mm trocar, supraumbilical | 8 mm trocar | 8 mm trocar |  | 12 mm trocar, above the umbilicus | 5 mm trocar, below the umbilicus |  |  |  |  |  |  |
| Wang et al., 2022 | KangDuo |  |  |  |  |  |  | 12 mm trocar, at the intersection of the midaxillary line and 2 cm below the costal margin | 10 mm trocar, at the costal ridge angle | 10 mm trocar, at the intersection of the anterior axillary line and the subcostal margin |  | 12 mm trocar on the dorsal side | 5 mm trocar on the ventral side |
| Meneghetti et al., 2024 | Versius | 12 mm trocar, 2 cm lateral to the umbilicus | 5 mm trocar, 12 cm from the optical trocar in the direction of 4 cm cranial to the ipsilateral anterosuperior iliac spine | 5 mm trocar, 12 cm from the optical trocar in the direction of the patient's shoulder |  | 10 mm assistant trocar, 8 cm equidistant from the Maryland trocar and the camera trocar | 5 mm trocar for right sided tumors |  |  |  |  |  |  |
| Gupta et al., 2025a | KangDuo/EDGE | 10 mm trocar, at the outer border of rectus abdominis, supraumbilical | At the anterior axillary line under the umbilicus | Midclavicular line of the affected side, 1 cm below the costal edge |  | A 12 mm trocar 5 cm above the umbilicus | 12 mm trocar 5 cm below the umbilicus | 10 mm trocar, 2 cm below costal border at mid-axillary line | 10 mm trocar, at the costal ridge angle | At the anterior axillary line |  | 12 mm trocar on the ventral side |  |
| Chen et al., 2025 | Toumai | 12 mm trocar, 2 cm upper to the umbelicus | 8 mm trocar, on the pararectal line | 8 mm trocar, 2 cm below the xifoid process | 8 mm trocar, 2 cm below to the umbelicus | A 12 mm trocar, on the controlateral pararectal line |  |  |  |  |  |  |  |


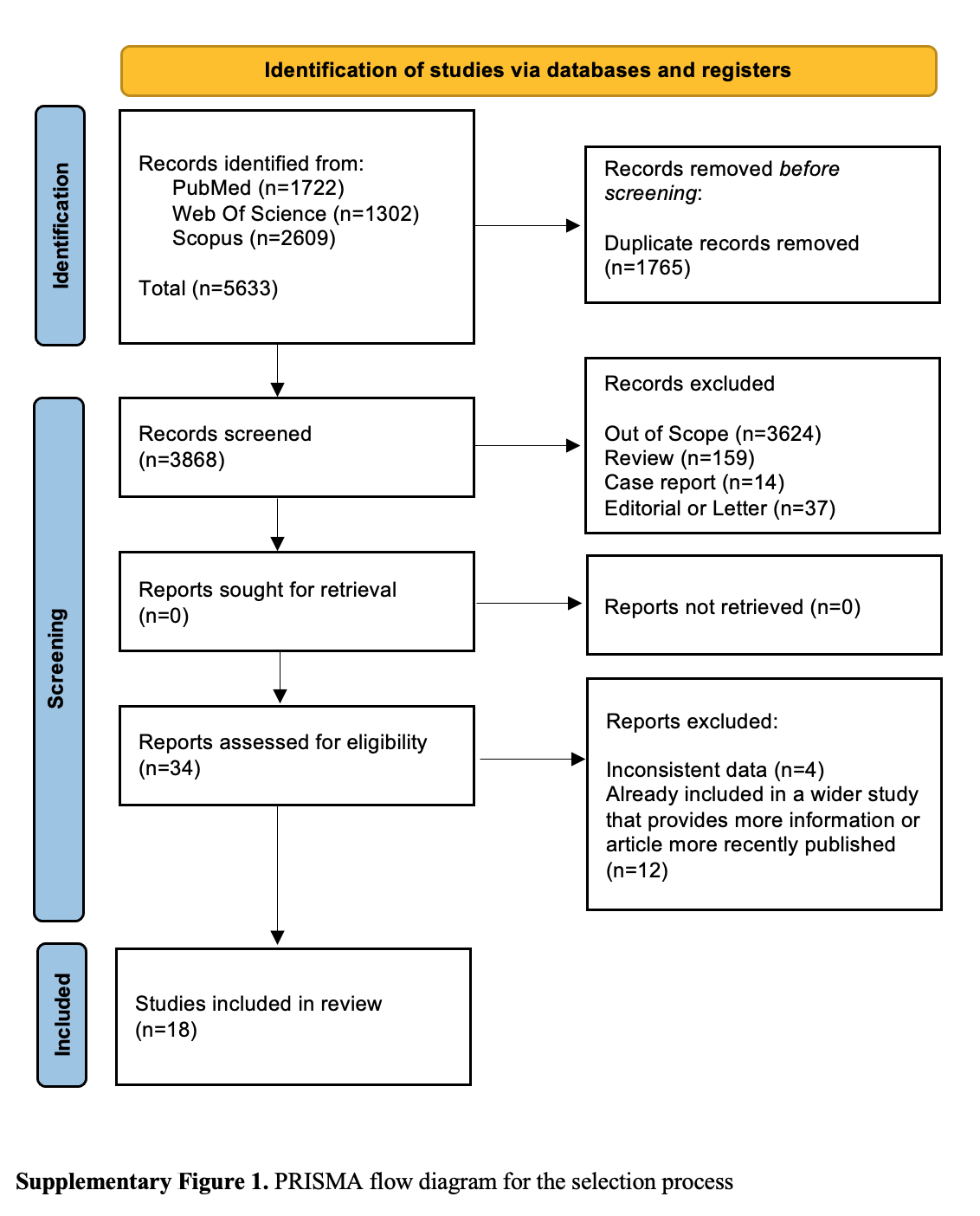


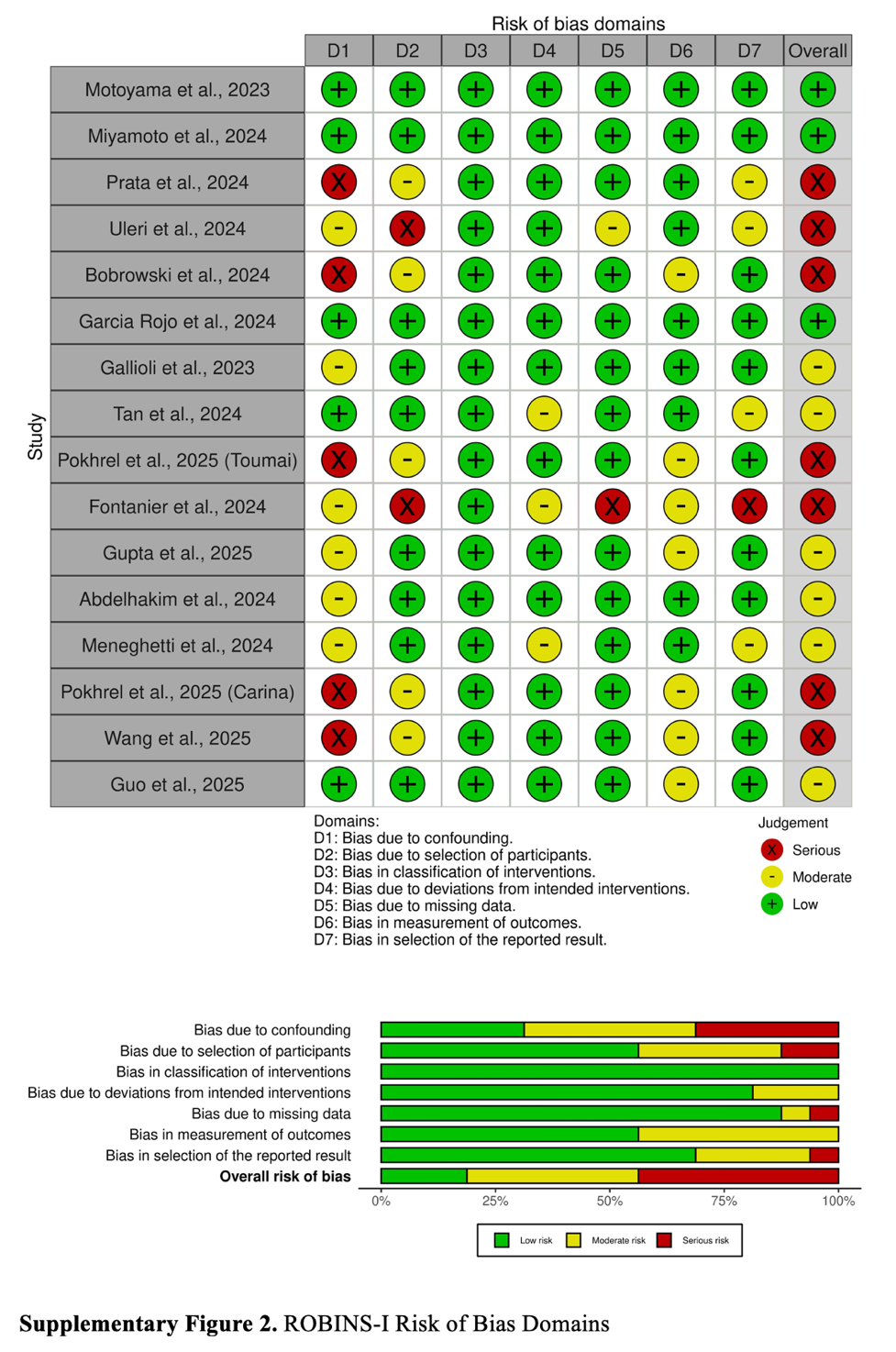


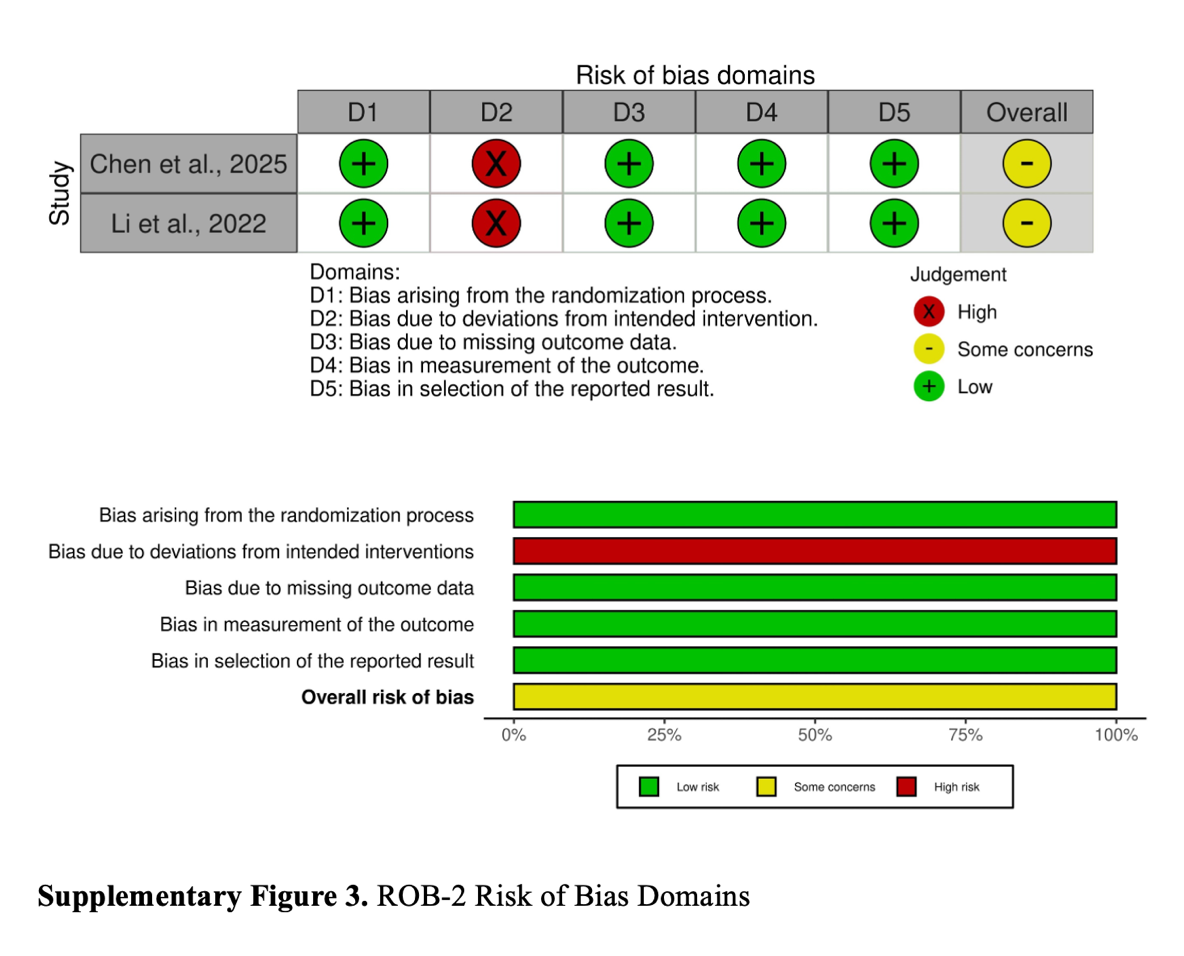


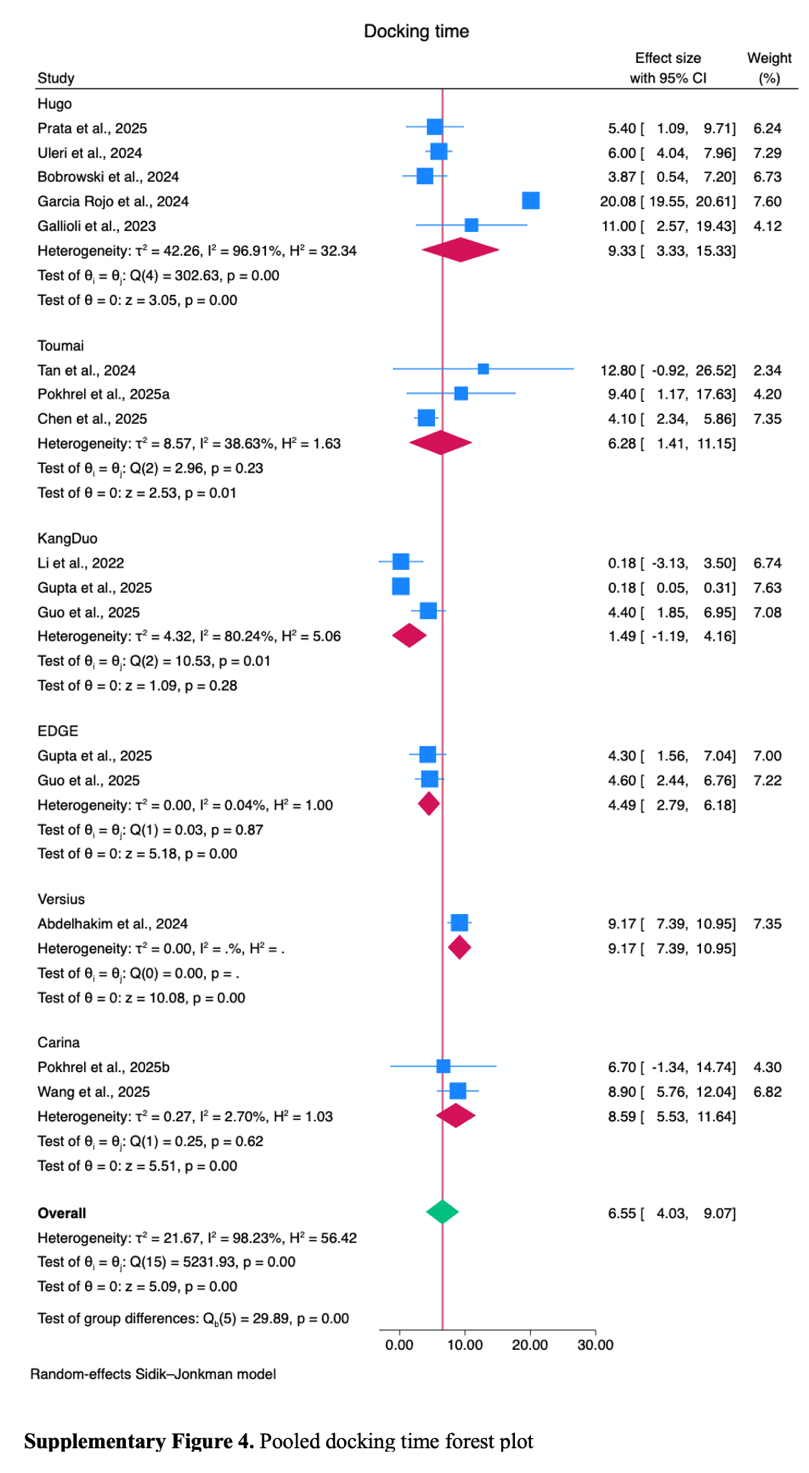

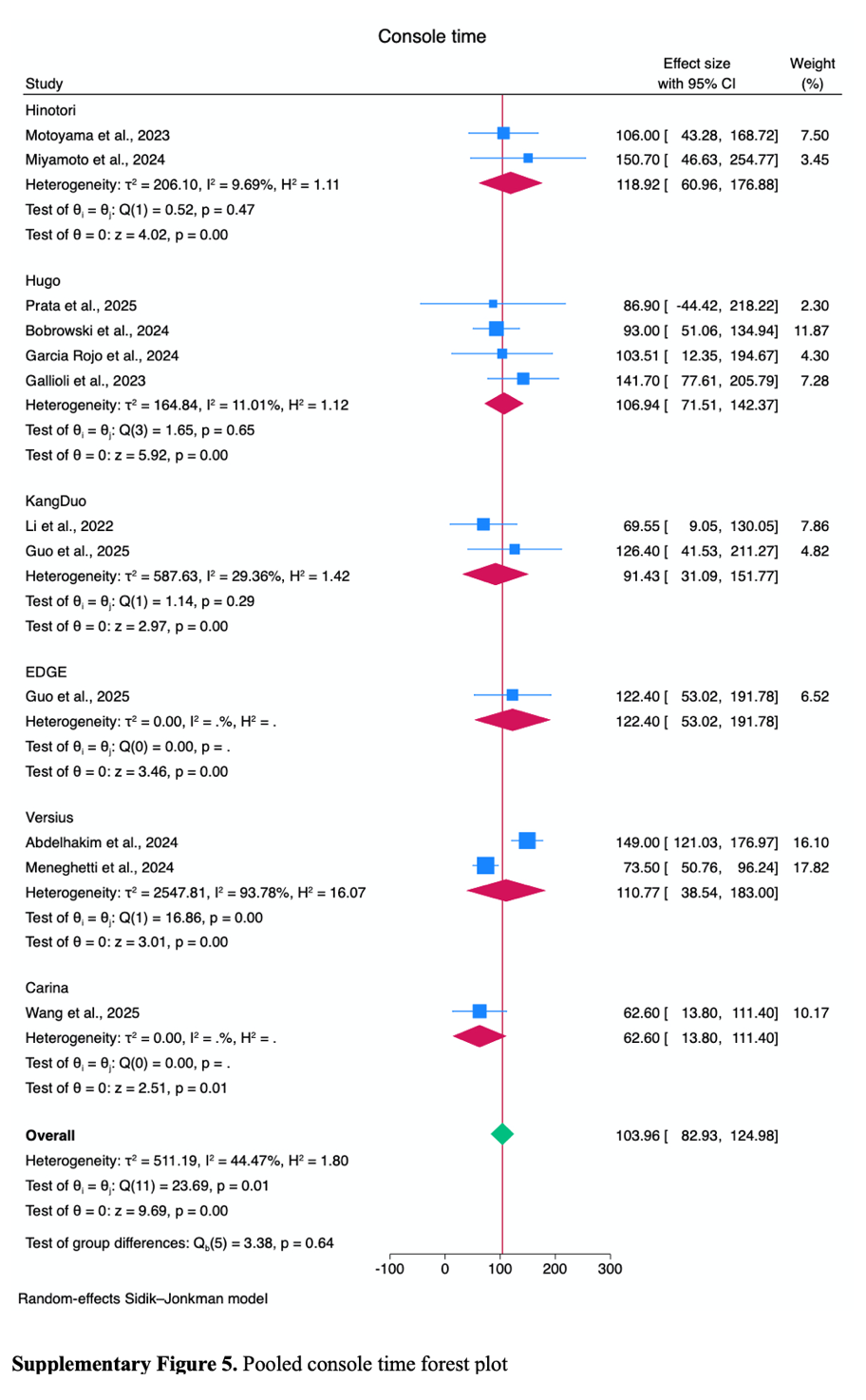


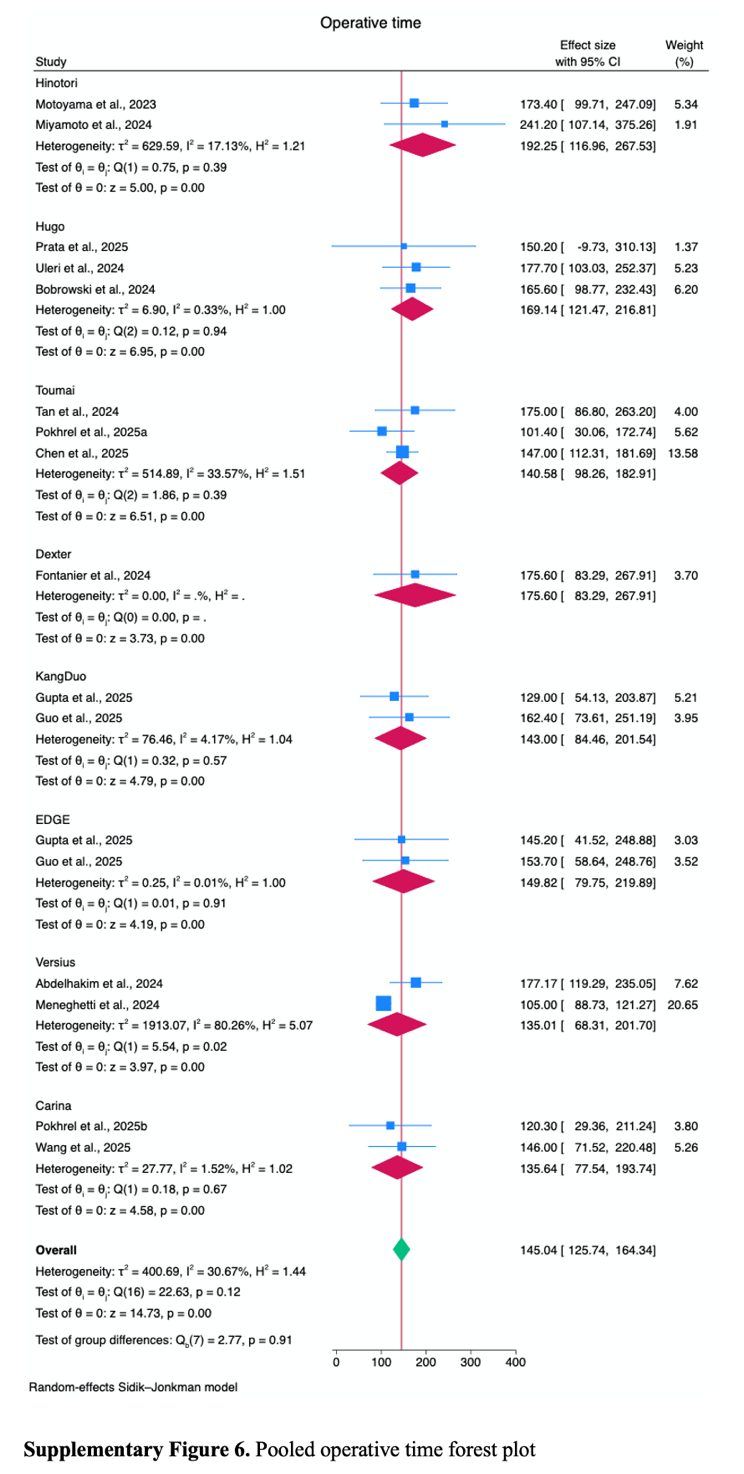

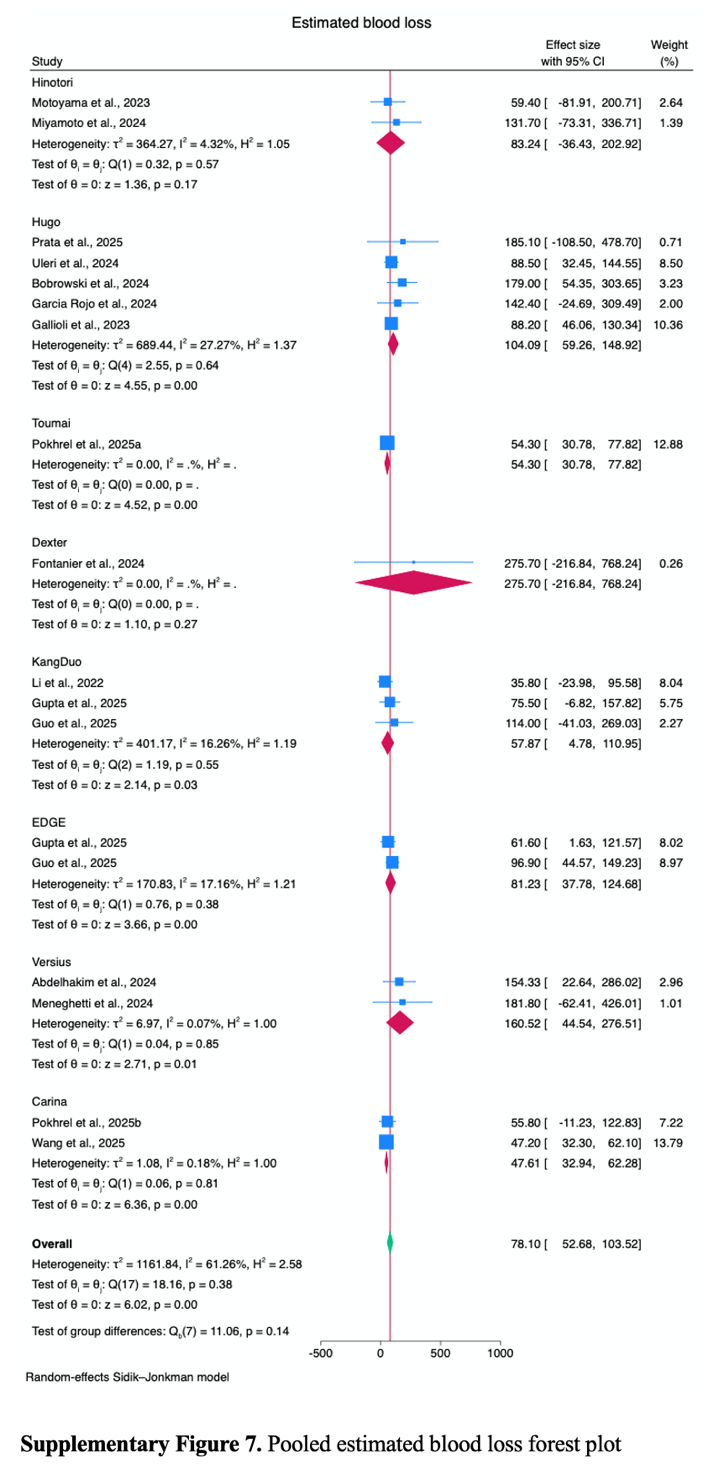

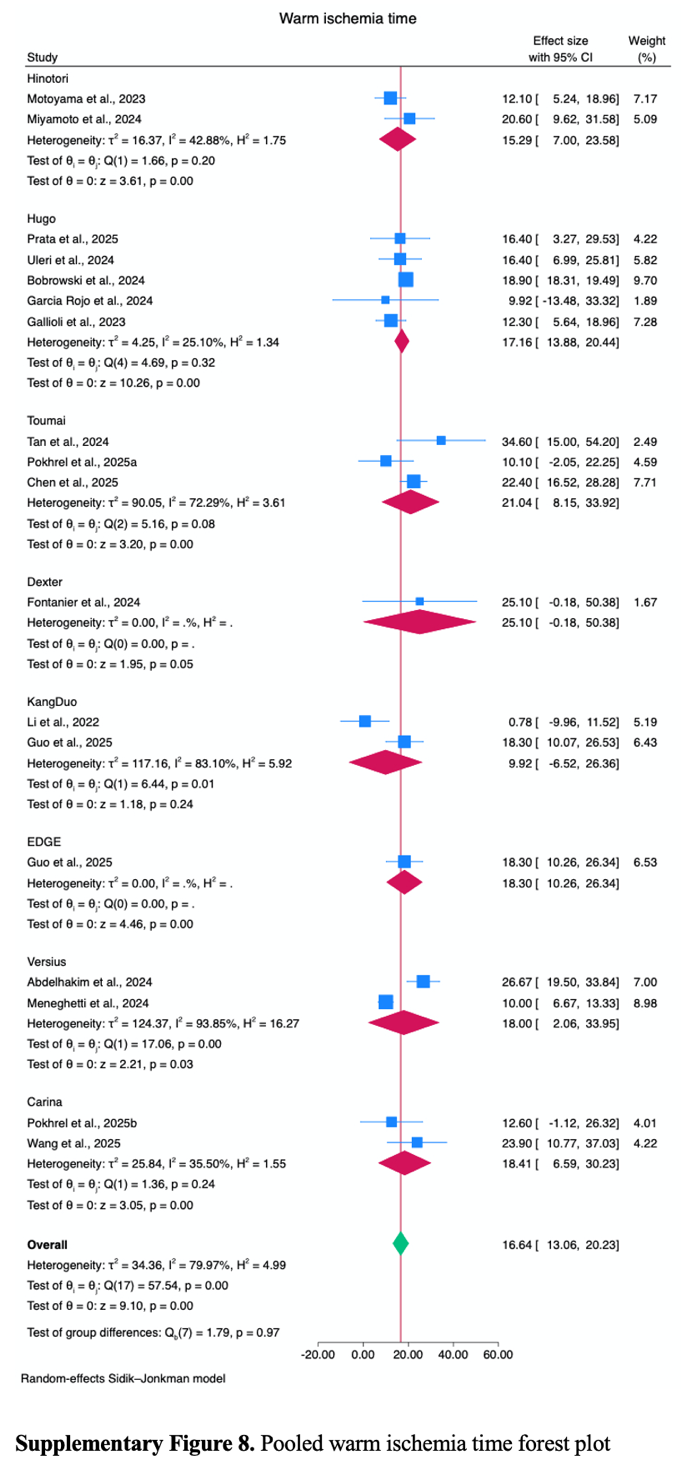

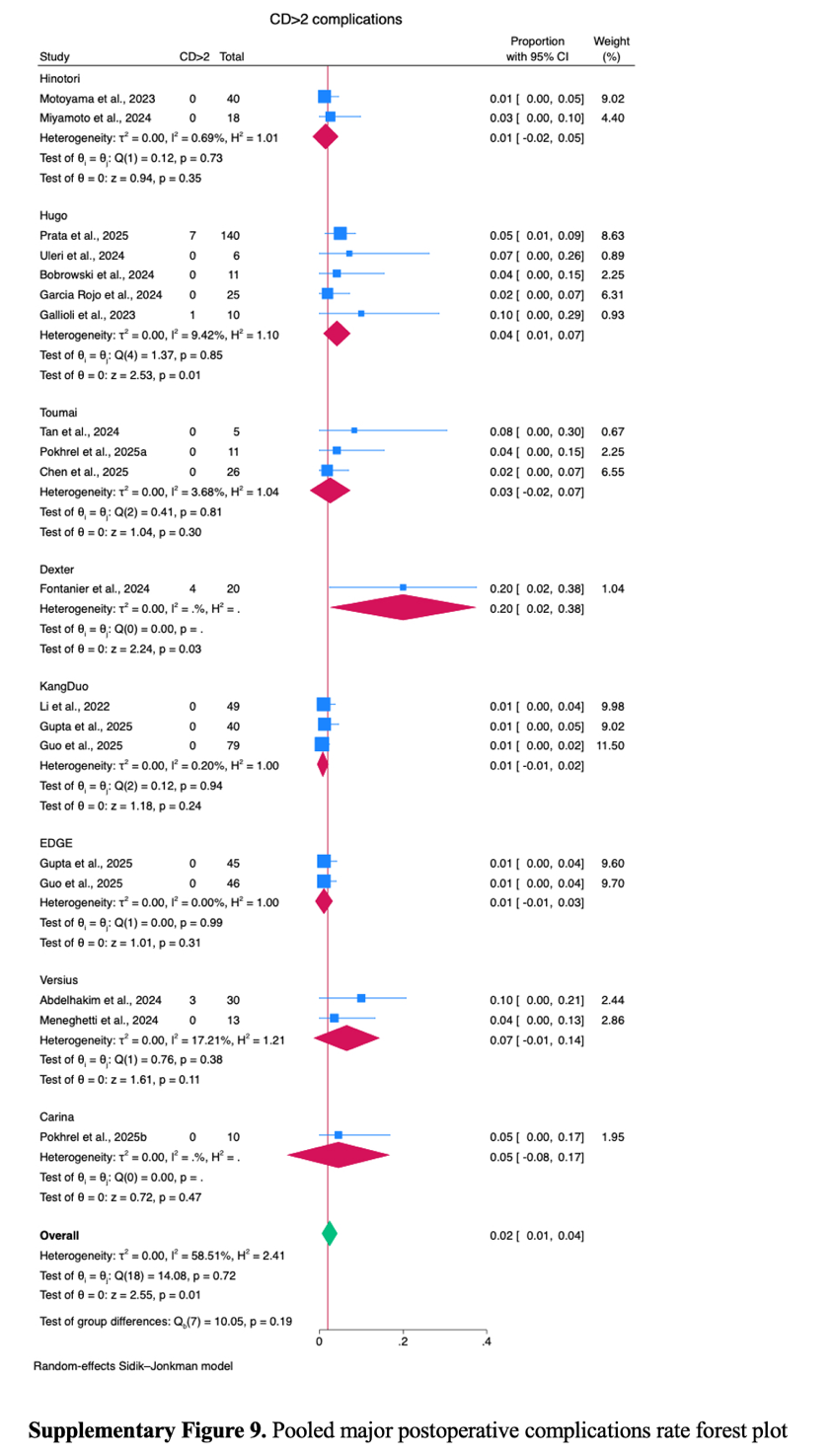

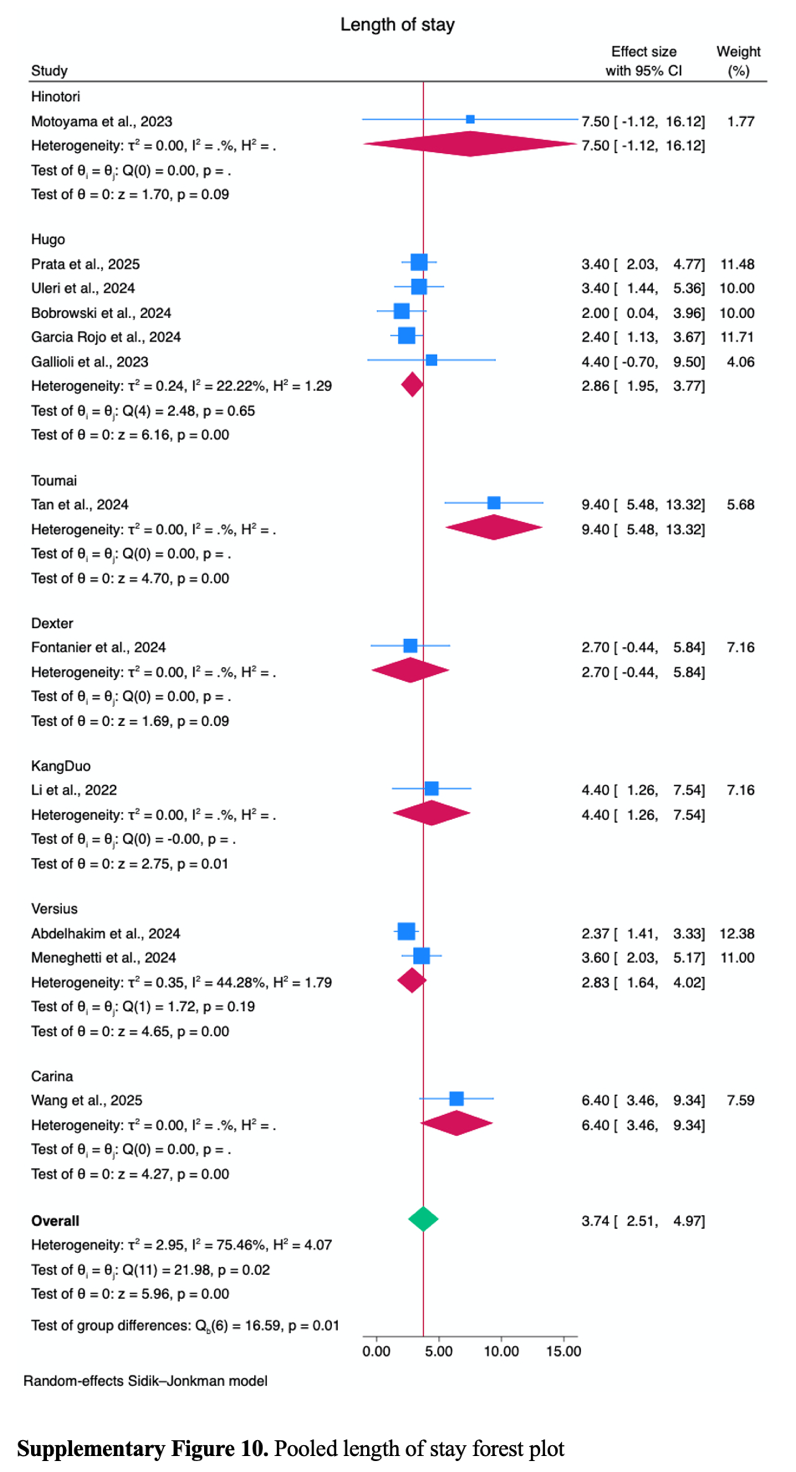

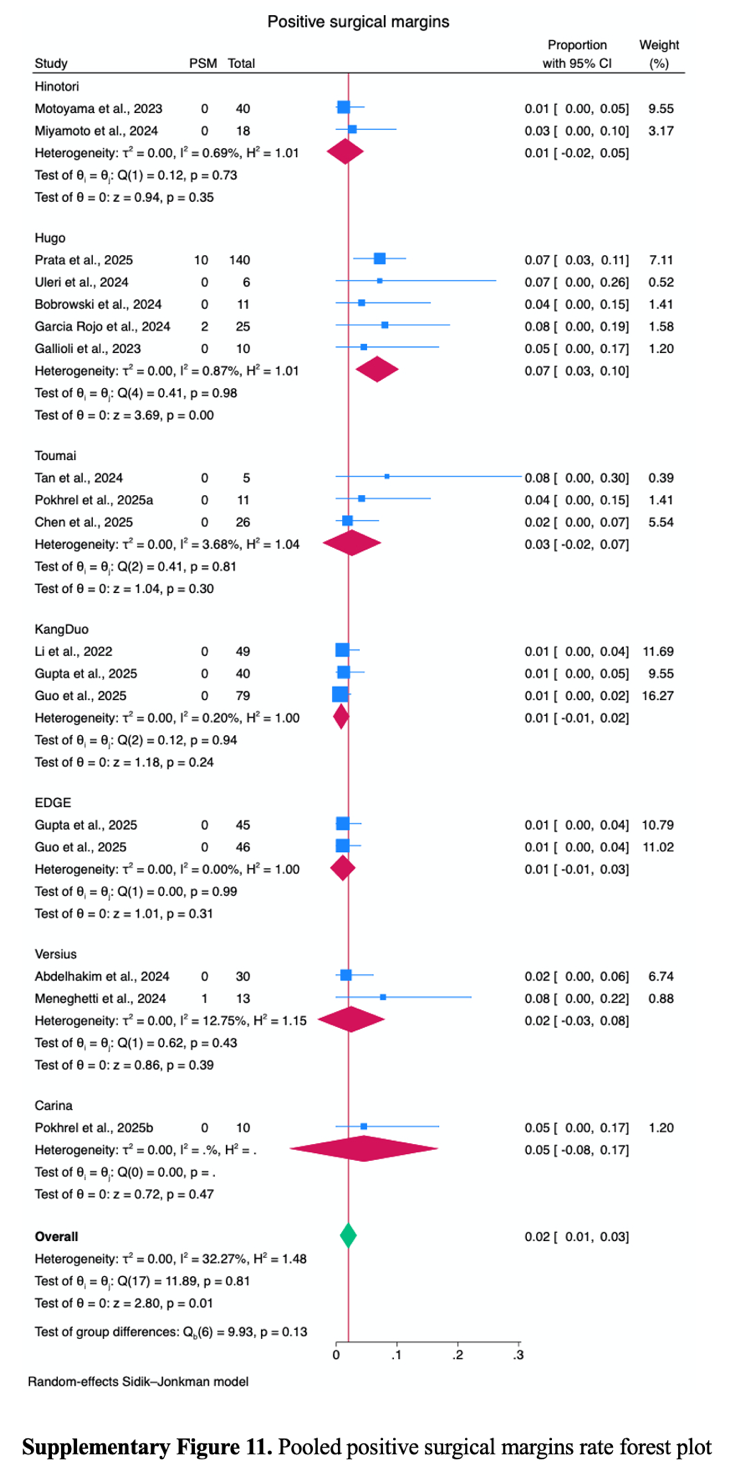

Supplement: Supplementary file 1 — Supplementary Material 1 [file 11701_2026_3277_MOESM1_ESM.docx]
